# Erucin Targets Oncogenic Signaling Pathways in Triple-Negative Breast Cancer: An Integrated Network Pharmacology and In Vitro Study

**DOI:** 10.3390/life16050708

**Published:** 2026-04-22

**Authors:** Humera Banu, Eyad Al Shammari, Husam Qanash, Mitesh Patel, Mohd Adnan, Syed Shahanawaz, Mohammad Idreesh Khan, Malak Yahia Qattan, Syed Amir Ashraf

**Affiliations:** 1 Department of Clinical Nutrition, College of Applied Medical Sciences, University of Ha’il, Ha’il P.O. Box 2440, Saudi Arabia; 2 Department of Medical Laboratory Science, College of Applied Medical Sciences, University of Ha’il, Ha’il 55476, Saudi Arabia; 3 Medical and Diagnostic Research Center, University of Ha’il, Ha’il 55473, Saudi Arabia; 4 Department of Bioinformatics, Faculty of Engineering and Technology, Marwadi University, Rajkot 360003, Gujarat, India; 5 Department of Biology, College of Science, University of Ha’il, Ha’il P.O. Box 2440, Saudi Arabia; 6 Department of Physical Therapy, College of Applied Medical Sciences, University of Ha’il, Ha’il P.O. Box 2440, Saudi Arabia; 7 Department of Basic Health Sciences, College of Applied Medical Sciences, Qassim University, P.O. Box 6666, Buraydah 51452, Saudi Arabia; 8 Department of Health Sciences, College of Applied Studies, King Saud University, P.O. Box 4545, Riyadh 11451, Saudi Arabia

**Keywords:** apoptosis, cell cycle arrest, *Eruca sativa*, erucin, isothiocyanates, MDA-MB-231 cells

## Abstract

This study aims to investigate the potential anticancer effects of erucin, an isothiocyanate derived from *Eruca sativa*, in triple-negative breast cancer (TNBC) by predicting molecular targets and evaluating its in vitro effects on TNBC cell proliferation, apoptosis and cell cycle distribution. Potential protein targets of erucin were identified using SwissTargetPrediction, resulting in 117 targets, of which 84 overlapped with TNBC-related genes sourced from GeneCards, DisGeNET, and OMIM. Protein–protein interaction analysis was performed to identify key hub genes. In vitro experiments were conducted using MDA-MB-231 TNBC cells to assess dose-dependent effects on cell viability. Flow cytometry was employed to evaluate apoptotic cell populations and cell cycle distribution. Protein–protein interaction analysis identified ten hub genes, including AKT1, STAT3, EGFR, and MMP9, representing highly connected nodes within the predicted interaction network. In vitro studies showed dose-dependent reduction in MDA-MB-231 cell viability following erucin treatment, with an IC_50_ of approximately 48.87 µg/mL. Flow cytometry revealed increased apoptotic cell population and G1 phase accumulation. These findings suggest that erucin is associated with cytotoxic and antiproliferative effects in TNBC cells and may interact with multiple cancer-related targets. However, the identified molecular targets and pathways are based on computational predictions and require further experimental validation. Overall, this study provides a preliminary integrated framework linking computational predictions with experimental observations, which may support future mechanistic and preclinical investigations of erucin in TNBC.

## 1. Introduction

Triple-negative breast cancer (TNBC) is an aggressive subtype of breast cancer characterized by the lack of expression of estrogen receptors (ER), progesterone receptors (PR), and human epidermal growth factor receptor 2 (HER2). It accounts for approximately 10–20% of breast cancer cases and is more frequently observed in younger women, particularly those with BRCA1 mutations, as well as in African, American and Hispanic populations. TNBC poses significant clinical challenges due to the lack of targeted therapeutic options and its association with poor prognosis, high recurrence rates and therapeutic resistance [1,2]. Recent research has uncovered the molecular heterogeneity of TNBC, illustrating the heterogeneity in gene expression that influences prognosis and therapeutic response [3]. Specific genes, such as *TMPRSS7* and *PGM2L1*, have been proposed as potential prognostic markers, offering valuable insight into patient stratification and personalized treatment planning [4]. Despite advances in conventional treatments such as surgery, chemotherapy, and radiotherapy, long-term disease management remains limited by resistance and adverse effects [5,6].

In this context, there is increasing interest in identifying novel bioactive compounds with improved efficacy and reduced toxicity. Naturally derived compounds from dietary and medicinal plants have attracted attention due to their ability to modulate multiple biological processes involved in cancer progression. Various classes of phytochemicals, including flavonoids, alkaloids, terpenoids and sulfur-containing compounds, have demonstrated anticancer potential in experimental models by inducing apoptosis, regulating cell cycle progression and inhibiting metastasis [7,8]. However, the precise molecular interactions and multi-target effects of many such compounds remain incompletely understood.

Among sulfur-containing compounds, sulforaphane and its structural analog erucin have shown promising anticancer activities. Erucin, abundantly present in cruciferous vegetables such as arugula and broccoli, has been reported to exhibit antioxidant, anti-inflammatory and antiproliferative effects [7,9]. It shares structural similarities with sulforaphane and has been found to target cancer hallmarks by inhibiting cell proliferation, inducing apoptosis and altering cell cycle regulation [7]. Evidence suggests that erucin activates autophagic and apoptotic pathways, thereby inhibiting tumor growth and metastasis [5]. Moreover, computational approaches highlighted its high binding affinity to key oncogenic targets, supporting its potential utility in cancer therapy [7]. Notably, erucin is reported to trigger autophagy-dependent apoptotic cell death in TNBC cells, confirming its anticancer relevance in this specific subtype [7]. In addition, computational studies have suggested potential interactions of erucin with oncogenic targets. However, a comprehensive systems-level understanding of its potential multi-target interactions in TNBC remains limited.

Therefore, the present study is designed to address this gap by employing an integrative strategy that combines network pharmacology-based target prediction and molecular docking analyses with in vitro cytotoxicity testing, apoptosis, and cell cycle assays in MDA-MB-231 cells. Unlike studies focusing on single pathways, this approach enables the identification of potential target networks and associated biological processes. The findings of this study are intended to provide a preliminary systems-level framework that may guide future mechanistic and preclinical investigations of erucin in TNBC [9].

## 2. Materials and Methods

### 2.1. Identification of Potential Targets for Associated Pathologies

The SMILES representation of erucin was obtained from the PubChem database. To predict its potential biological targets, this SMILES code was submitted to the SwissTargetPrediction tool (www.swisstargetprediction.ch, accessed on 23 March 2025). A comprehensive set of genes linked to TNBC was extracted from the GeneCards platform (https://www.genecards.org, accessed on 23 March 2025) using the query “triple-negative breast cancer.” Further disease-relevant genes were curated from DisGeNET (http://www.disgenet.org, accessed on 23 March 2025) and OMIM (https://www.omim.org, accessed on 23 March 2025). For inclusion, genes from DisGeNET had to meet a gene-disease association (GDA) score above 0.1 and those from GeneCards required a relevance score greater than 30 [10]. The GeneCards relevance score threshold of >30 was applied to retain genes with moderate-to-strong evidence of association derived from text-mining, curated databases, and experimental data. The DisGeNET GDA score threshold of >0.1 was used to exclude weakly supported gene–disease associations, retaining only those with meaningful curated evidence. These thresholds are consistent with widely adopted practices in network pharmacology studies [10,11].

### 2.2. Identification and Acquisition of Common Targets

To determine overlapping targets between erucin and TNBC, the FunRich software (version 3.1.3) was used. A Venn diagram was constructed to identify shared targets between the compound and disease datasets [12].

### 2.3. Hub Gene Identification and Protein Interaction Network Construction

Key hub genes were identified using the cytoHubba plugin within Cytoscape (version 3.10.1), employing a degree-based algorithm to extract the top ten most connected nodes. To assess interactions between target proteins, the STRING database (https://string-db.org, accessed on 24 March 2025) was utilized [13]. A false discovery rate (FDR) threshold of 5% and a confidence score cutoff of 0.400 were applied during data filtering. The PPI networks imported into Cytoscape were analyzed using three centrality measures, including degree, betweenness and closeness, to prioritize influential targets within the network.

### 2.4. Enrichment Analysis of GO and KEGG Pathways

Functional annotation and pathway enrichment of the identified hub genes were performed using the DAVID platform (https://davidbioinformatics.nih.gov/, accessed on 24 March 2025) [14]. Statistical significance was determined using a false discovery rate (FDR) threshold of <0.05. The top ten enriched Gene Ontology (GO) terms across the Biological Process (BP), Cellular Component (CC), and Molecular Function (MF) categories were visualized via the SRplot online tool. The KEGG pathway enrichment results were further explored using ShinyGo version 0.77 [15]. These analyses were performed to identify potential biological processes and pathways associated with the predicted targets.

### 2.5. Molecular Docking

Molecular docking was executed using PyRx 0.9.8, which integrates AutoDock Vina v1.2.0 to estimate binding affinities between erucin and target proteins. Three top-ranked targets, AKT1 (PDB ID: 8UW7), STAT3 (PDB ID: 6NUQ) and PTGS2 (PDB ID: 5KIR were selected. Their crystal structures were processed and used as receptor molecules. The erucin ligand was minimized for energy and converted to PDBQT format. Docking grids were defined to enclose known binding pockets using specific center coordinates and box dimensions (Table 1). Docking was performed with an exhaustiveness level of 8. The best-scoring binding poses were selected for interaction analysis to explore potential ligand–protein interactions.

### 2.6. Cell Culture

The MDA-MB-231 cell line was procured from the National Centre for Cell Science (NCCS), Pune, India. Cells were cultured in Dulbecco’s Modified Eagle Medium (DMEM) supplemented with 10% fetal bovine serum (FBS), 10,000 units/mL penicillin, and 5 mg/mL streptomycin, and maintained at 37 °C in a humidified incubator with 5% CO_2_ [16].

### 2.7. Cell Viability Assay

The MTT assay was utilized to assess cytotoxicity. This assay provides a measure of cell metabolic activity as an indicator of cell viability. MDA-MB-231 cells were harvested from T-25 flasks by trypsinization, pelleted by centrifugation at 1000× *g* for 5 min, and resuspended in fresh medium at a density of 10,000 cells per 200 µL. This suspension was seeded into 96-well plates and incubated for 24 h at 37 °C to facilitate adhesion. Following medium aspiration, cells were exposed to erucin at concentrations of 1, 10, 100, 250, 500, and 1000 µg/mL for an additional 24 h. Post-treatment, 10% MTT reagent was added and incubated for 3 h, after which 100 µL of DMSO was introduced to solubilize formazan crystals. Absorbance was recorded at 570 nm and 630 nm, and the IC_50_ value of erucin was calculated to evaluate its antiproliferative efficacy against MDA-MB-231 cells [17]. All experiments were performed in triplicate across three independent biological replicates.

### 2.8. Annexin-V Apoptosis Assay

MDA-MB-231 cells were plated at a density of 5 × 10^4^ cells/well in 6-well culture plates and allowed to adhere for 24 h. The cells were then exposed to erucin at the predetermined IC_50_ concentration for 24 h. This assay was conducted to assess apoptosis induction following erucin treatment. After rinsing with phosphate-buffered saline (PBS), dual staining with Annexin V-FITC and PI was performed per standard procedures, and apoptotic populations were quantified by flow cytometry using the BD FACS Lyric™ system (BD Biosciences, Franklin Lakes, NJ, USA) [18]. The assay was conducted in three independent experiments (n = 3).

### 2.9. Cell Cycle Analysis

MDA-MB-231 cells were plated and treated with erucin (IC_50_) in 6-well plates as outlined above. Cell cycle analysis was performed to evaluate whether erucin disrupts cell cycle progression, as predicted from the enrichment of proliferation-related pathways in the network pharmacology analysis. Post-treatment, cells were trypsinized, fixed in ice-cold 70% ethanol, and stained with a PI staining buffer supplemented with RNase A and Triton X-100. The proportion of cells in G1, S, and G2/M phases was determined using flow cytometric analysis of DNA content [19]. All experiments were conducted in triplicate (n = 3).

### 2.10. Statistical Analysis

MTT assay data are expressed as mean ± SD across three biological replicates. Group comparisons were performed using one-way ANOVA followed by Dunnett’s multiple comparison test, with statistical significance defined at *p* < 0.05. Analyses were executed in the GraphPad Prism software, version 8.0.

## 3. Results

### 3.1. Target Prediction and Identification of Potential Overlapping Genes

**Figure 1:**
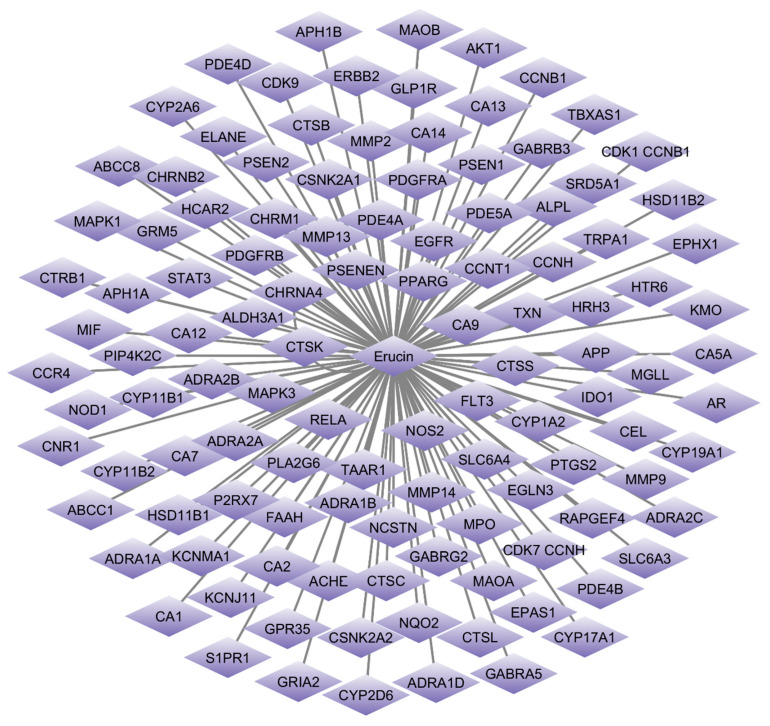
Erucin–target protein interaction network after removal of duplicate protein targets. The network illustrates the interactions between erucin (central purple node) and its associated putative target proteins (purple diamond-shaped nodes). The nodes represent erucin and individual target proteins, while the edges (gray lines) denote the predicted interactions between Erucin and these protein targets.

**Figure 2:**
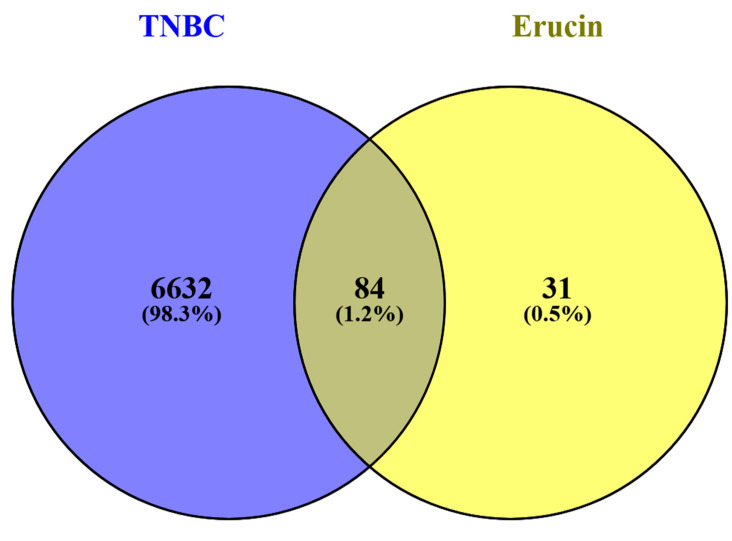
Venn diagram showing overlapping targets between erucin and triple-negative breast cancer (TNBC).

SwissTargetPrediction was utilized to identify the potential protein targets for erucin. A total of 117 potential targets were identified through this tool. Concurrently, TNBC-related gene data were compiled from GeneCards (GDA cutoff > 30), DisGeNET (cutoff > 0.1) and OMIM databases. After removing duplicate entries, 6717 genes associated with TNBC were retained. Venn diagram analysis using FunRich revealed 84 common genes shared between erucin targets and TNBC-associated genes (Figure 1 and Figure 2), indicating a potential overlap between predicted compound targets and disease-associated genes.

### 3.2. Hub Gene Detection and Network Construction of Compound-Disease Targets

**Figure 3:**
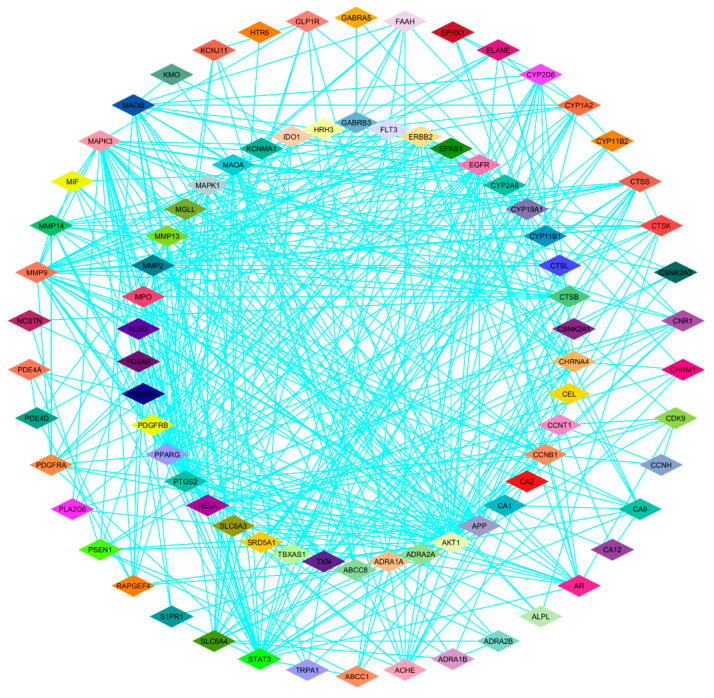
Network of common target proteins between erucin and triple-negative breast cancer (TNBC). The protein–protein interaction (PPI) network illustrates the shared targets between Erucin and TNBC. Each node represents a common protein target, while the edges (light blue lines) denote predicted or known interactions among these targets. Nodes are color-coded based on cluster groupings or functional modules identified via network topology analysis, highlighting the complex connectivity and potential signaling pathways modulated by erucin in the context of TNBC.

**Figure 4:**
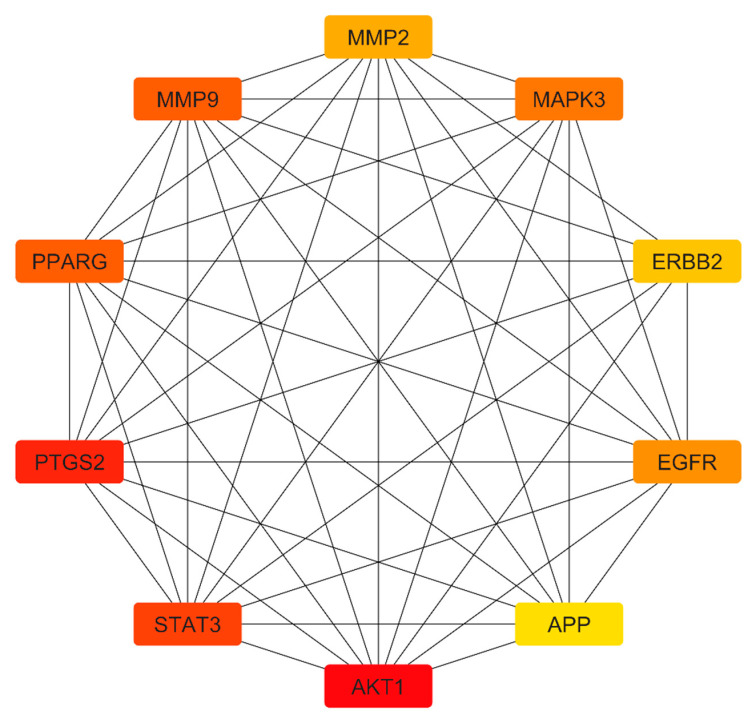
Protein–protein interaction (PPI) network of hub target genes shared between erucin and triple-negative breast cancer (TNBC). The network visualizes the top hub genes among the common targets of Erucin and TNBC, identified through topological analysis of the larger protein interaction network. Nodes represent individual proteins, with node color intensity reflecting degree centrality red indicating higher connectivity and potential biological importance. The edges denote functional or physical associations between the proteins. Key hub targets include AKT1, STAT3 and PTGS2 highlighting critical nodes likely contributing to therapeutic mechanisms of erucin in TNBC.

To identify key regulatory genes, a protein–protein interaction (PPI) network was generated using the STRING database. Network visualization and topological analysis were performed using the Cytoscape software (version 3.10.1) (Figure 3). Using the cytoHubba plugin and a degree-based ranking method, ten hub genes with high connectivity were identified as *AKT1*, *PTGS2*, *STAT3*, *PPARG*, *MMP9*, *MAPK3*, *EGFR*, *MMP2*, *ERBB2* and *APP* (Figure 4). These genes represent highly connected nodes within the network and may be associated with TNBC-related biological processes.

### 3.3. Functional Annotation and Pathway Enrichment Analysis

**Figure 5:**
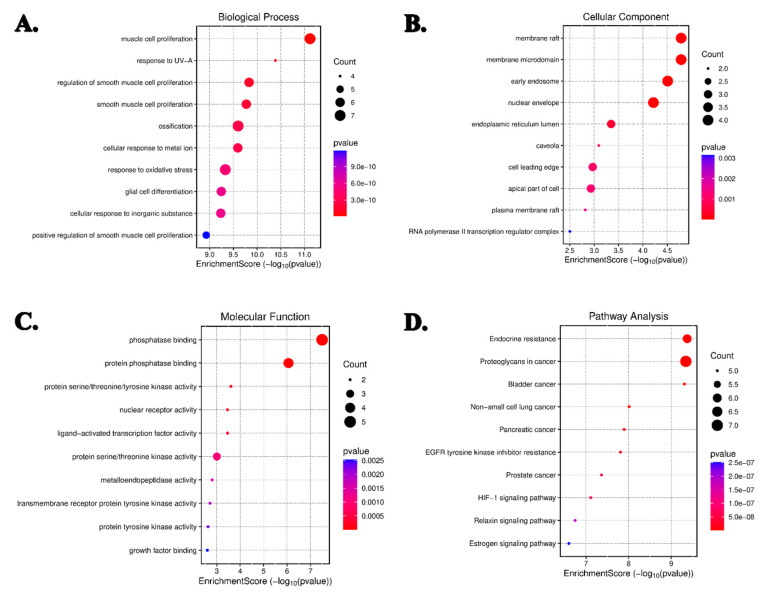
KEGG pathway and GO enrichment analyses of identified apoptosis target proteins (*p*-value ≤ 0.05). (**A**) Top 10 biological processes, (**B**) Top 10 cellular processes, (**C**) Top 10 molecular functions, and (**D**) Top 10 KEGG pathways.

To delineate the biological roles of the identified target genes, enrichment analysis was performed using the DAVID platform. Gene Ontology (GO) classification assigned the genes to 1474 biological processes (BPs), 102 cellular components (CCs), and 109 molecular functions (MFs). The most significantly enriched terms included responses to UV-A radiation, oxidative stress, smooth muscle cell proliferation, ossification, and metal ion stimuli. Furthermore, 154 KEGG pathways were associated with these genes, with prominent enrichment observed in EGFR tyrosine kinase inhibitor resistance, HIF-1 signaling, estrogen signaling, and proteoglycans in cancer pathways (Figure 5A–D). These enrichment results suggest that the predicted targets are associated with multiple cancer-related biological processes and pathways.

### 3.4. Molecular Docking

**Figure 6:**
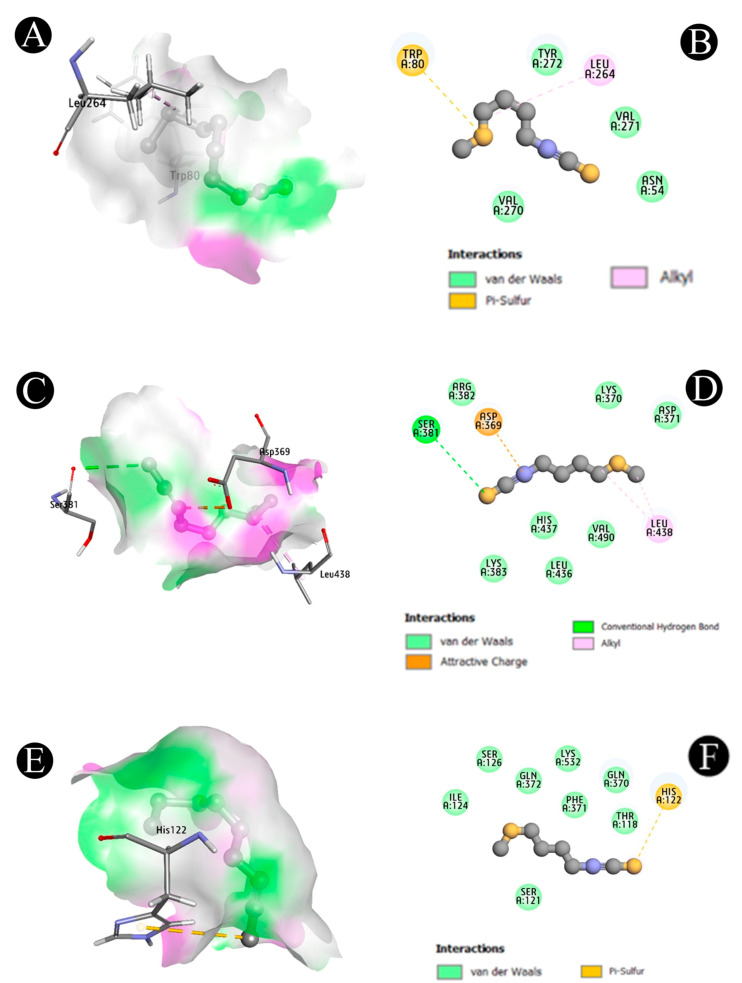
Molecular Docking Analysis of erucin with target proteins. (**A**,**B**). 3D and 2D visualizations of the interaction between erucin and AKT1, highlighting key binding residues and interaction types, (**C**,**D**). 3D and 2D visualizations of the interaction between erucin and STAT3, showing the docking pose and molecular interactions within the binding pocket, (**E**,**F**). 3D and 2D visualizations of the interaction between erucin and PTGS2, illustrating the binding orientation and involved amino acid residues.

Docking analysis were conducted to assess the binding affinity of erucin with three TNBC hub targets, including AKT1 (8UW7), STAT3 (6NUQ) and PTGS2 (5KIR). Discovery Studio Visualizer v2020.1 was used to analyze molecular interactions. Erucin exhibited favorable binding affinities as −7.5 kcal/mol with AKT1, −8.2 kcal/mol with STAT3 and −7.4 kcal/mol with PTGS2. Specific interactions included hydrogen bonding with residues such as LEU 264, VAL 270 and TYR 272 in AKT1, as well as ASP 369 and HIS 437 in STAT3. Pi-sulfur interactions were observed between erucin and HIS 122 in PTGS2. These computational interactions suggest that erucin may potentially bind and modulate key proteins implicated in TNBC (Table 2; Figure 6A–F). These findings indicate favorable binding interactions between erucin and the selected target proteins, supporting potential ligand–protein associations.

### 3.5. Cytotoxic Effects of Erucin on TNBC Cells

**Figure 7:**
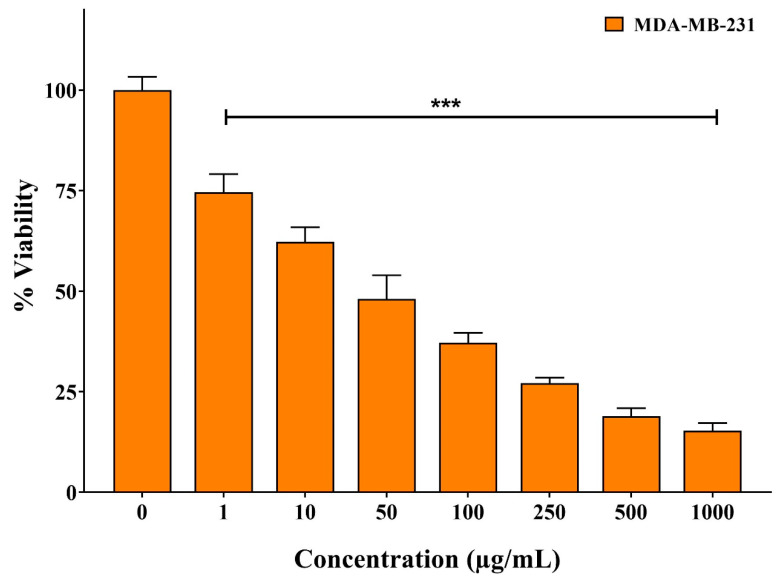
Quantitative cytotoxicity analysis of erucin on MDA-MB-231 cells using MTT assay. The graph illustrates a dose-dependent decrease in cell viability following treatment with various concentrations of erucin. Cell viability is expressed as a percentage relative to the untreated control group (set at 100%). Data are presented as the mean ± SD from three independent experiments (n = 3). Statistical significance was evaluated using one-way ANOVA followed by Dunnett’s multiple comparison test against the untreated control: *** *p* < 0.0005.

The antiproliferative effect of erucin on MDA-MB-231 TNBC cells was evaluated using the MTT assay. Cell viability was inhibited in a dose-dependent manner, yielding an IC_50_ of 48.87 μg/mL, defined as the concentration required to suppress cell survival by 50%. Higher concentrations of erucin were associated with a significant reduction in cell viability, indicating its cytotoxic effect under the tested conditions (Figure 7).

### 3.6. Apoptosis Detection by Annexin V-FITC/PI Staining

**Figure 8:**
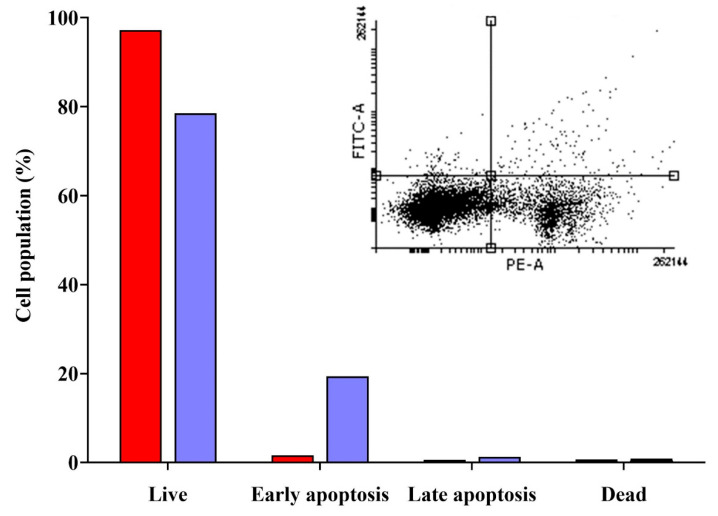
Assessment of apoptosis induction in MDA-MB-231 cells treated with erucin. Cells were treated with the IC_50_ concentration of erucin for 24 h and analyzed using the Annexin V-FITC/PI assay. The **left panel** represents the untreated control and the **right panel** represents erucin-treated cells. Quadrant interpretation: lower-left (Annexin V−/PI−, live cells), lower-right (Annexin V+/PI−, early apoptotic cells), upper-right (Annexin V+/PI+, late apoptotic cells), and upper-left (Annexin V−/PI+, necrotic cells). The experiment was performed in triplicate (n = 3). Where red color bar indicate control sample and purple bar indicate treated sample.

To evaluate apoptosis induction, MDA-MB-231 cells were treated with erucin (IC_50_ concentration) and apoptosis was quantified via Annexin V-FITC/PI staining. Flow cytometry analysis showed that 19.40% of cells entered early apoptosis, 1.30% were in late apoptosis, and 0.80% underwent necrosis (Figure 8). These observations indicate that erucin treatment is associated with an increase in apoptotic cell populations, particularly in the early apoptotic phase.

### 3.7. Cell Cycle Arrest Analysis

**Figure 9:**
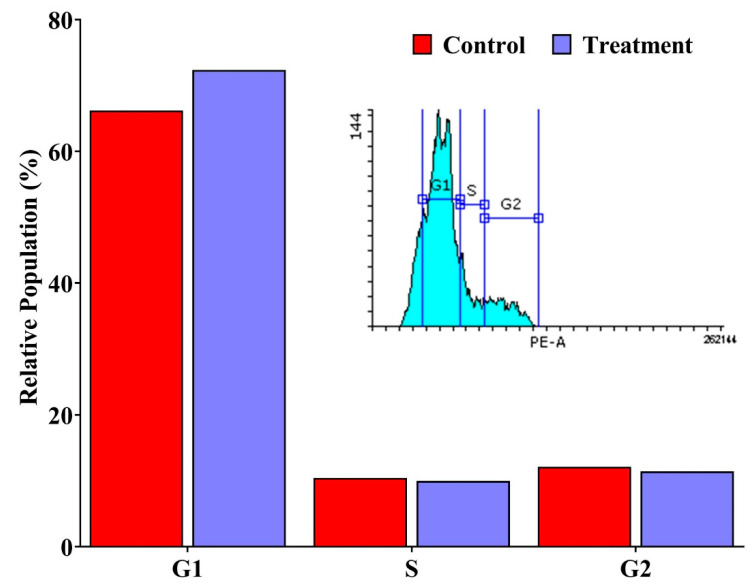
Cell cycle analysis of MDA-MB-231 cells treated with erucin. Cells were treated with the IC_50_ concentration of erucin for 24 h, followed by flow cytometric analysis of DNA content. The graph illustrates the mean percentage of cells in the G1, S, and G2/M phases for both control and erucin-treated groups. Data are presented as mean ± SD from three independent experiments (n = 3). Statistical comparisons between control and treated groups were performed using an unpaired Student’s *t*-test.

Flow cytometric evaluation of DNA content revealed that erucin treatment resulted in G1 phase accumulation in MDA-MB-231 cells, accompanied by a concomitant decrease in the proportions of cells in the S and G2/M phases (Figure 9). This shift in cell cycle distribution suggests that erucin treatment is associated with G1 phase accumulation in MDA-MB-231 cells.

## 4. Discussion

This study employed an integrated in silico and in vitro approach to investigate the potential anticancer effects of erucin, a naturally occurring isothiocyanate from *E. sativa*, against TNBC. Rather than focusing on a single pathway, the network pharmacology strategy adopted here enables a systems-level exploration of potential multi-target interactions, which distinguishes this work from prior studies that examined individual mechanisms of action.

Initial target predictions conducted using the SwissTargetPrediction database identified 117 potential protein targets for erucin. By integrating data from GeneCards, DisGeNET and OMIM, a comprehensive list of TNBC-associated genes was generated. Comparative analysis revealed overlapping targets, suggesting possible interactions between erucin and TNBC-associated proteins, consistent with previous reports on phytochemicals with multi-target effects [20,21]. This overlap highlights the role of erucin in targeting and possibly altering key signaling pathways relevant to TNBC. It is important to note that these targets are derived from computational prediction and represent candidate interactions requiring experimental validation.

The construction of a PPI network using the STRING database and Cytoscape software yielded ten hub genes with high connectivity including *AKT1*, *PTGS2*, *STAT3*, *PPARG*, *MMP9*, *MAPK3*, *EGFR*, *MMP2*, *ERBB2* and *APP*. These genes are known to be pivotal in cancer cell proliferation, survival, invasion and metastasis. Within the context of this study, these hub genes represent highly connected nodes that may be relevant to TNBC biology [22]. Understanding these network dynamics is essential, as demonstrated by reported studies that have highlighted similar gene interactions and their contributions to cancer biology. Furthermore, functional enrichment analysis utilizing the DAVID database revealed that the overlapping target genes are engaged in various biological processes pertinent to cancer progression, including pathways such as EGFR tyrosine kinase inhibitor resistance, HIF-1 signaling and estrogen signaling. The significant enrichment of these pathways suggests multifaceted effects of erucin on TNBC cells through interference with oncogenic signaling networks, aligning with findings from other natural compounds known to engage similar pathways [20]. These findings reflect statistical associations derived from enrichment analysis, and should be interpreted as indicative of potential pathway involvement rather than confirmed modulation.

In vitro assays demonstrated that erucin reduced MDA-MB-231 cell viability in a dose-dependent manner, with an IC_50_ value of approximately 48.87 μg/mL. Flow cytometric analysis employing Annexin V-FITC/PI staining indicated a substantial increase in early apoptotic cells post-erucin treatment, thereby confirming its role in apoptosis induction, a critical mechanism by which many anticancer agents exert their effects [23]. Additionally, cell cycle analysis demonstrated that erucin induces G1 phase arrest, hindering cell cycle progression and resulting in sustained cell proliferation inhibition. These observations indicate that erucin exerts measurable cytotoxic and antiproliferative effects under the tested conditions. While these cellular outcomes are consistent with processes such as apoptosis induction and cell cycle regulation, the present study does not establish direct pathway-specific mechanisms.

Bello et al. (2023) previously demonstrated that erucin triggers autophagy-dependent apoptotic cell death in TNBC cells, providing important evidence of erucin’s anticancer relevance [7]. The present study complements and extends those findings by adopting an unbiased network pharmacology approach that systematically maps the broader multi-target interaction network of erucin in TNBC. Specifically, the identification of hub genes such as *AKT1*, *STAT3*, and *PTGS2*, and the enrichment of pathways including EGFR-TKI resistance and HIF-1 signaling, provide a systems-level mechanistic context that was not addressed in the previous study. This integrated perspective offers a more comprehensive framework for understanding erucin’s potential mode of action in TNBC. Additionally, Ciccone et al. (2022) affirm that erucin can modulate inflammatory signaling pathways, further contributing to its anticancer properties. For instance, the reduction in COX-2 expression and inhibition of NF-κB activation by erucin can diminish inflammation and may directly affect the tumor microenvironment conducive to cancer progression [24].

The diverse biochemical actions of erucin extend to promoting cell cycle arrest and suppressing cellular proliferation. According to Singh et al., (2023), erucin is known to modulate phase I and phase III detoxification enzymes, supporting its role in reducing the viability of breast cancer cells [25]. Furthermore, Guerreiro et al. (2022) have reported that not only does erucin decrease cell viability in various cancer types, including breast cancer, but it also disrupts microtubule polymerization, which is crucial for cell division. In this context, the robust modulation of cellular processes by erucin makes it a candidate for further exploration in combination therapies aimed at enhancing the effectiveness of existing treatments [9]. Intriguingly, properties of erucin are enhanced when combined with other therapeutic agents. The findings by Kaur et al. (2022) suggest that a nanoemulsion formulation combining erucin and paclitaxel exhibits improved cytotoxicity against breast cancer cells compared to either compound alone. This synergy may offer a strategic approach to overcoming drug resistance often seen in breast cancer treatment [20]. Moreover, Martelli et al. (2021) highlight the adaptation of nanotechnology in enhancing the delivery and bioavailability of erucin, addressing the challenges associated with its pharmaceutical applications due to its poor solubility [26].

In addition to its direct anticancer effects, erucin also exhibits cardiovascular protective properties, which may indirectly benefit cancer patients by mitigating treatment-related cardiotoxicity. Martelli et al. (2021) elucidate how erucin reduces inflammation in endothelial cells, which could be beneficial for breast cancer patients undergoing chemotherapy, who often suffer from vascular damage. The dual role of erucin as both an anticancer agent and a cardioprotective compound exemplifies its potential as a versatile therapeutic agent in cancer treatment protocols [26]. Therefore, the evidence points to erucin as a promising candidate in the fight against breast cancer through multiple lymphatic and cellular mechanisms, including apoptosis induction, inflammation modulation, and synergistic effects when combined with existing chemotherapeutics. All these reported findings support the reproducibility of biological effects of erucin, while the current study extends this knowledge by integrating computational predictions with experimental observations.

Several limitations of this study should be acknowledged. First, the In vitro experiments were conducted using a single TNBC cell line (MDA-MB-231), which limits the generalizability of the findings across the heterogeneous spectrum of TNBC subtypes. Validation in additional TNBC cell lines (BT-549, MDA-MB-468) would strengthen the conclusions. Second, no in vivo experiments were performed; therefore, the translational relevance of these findings to animal models or clinical settings remains to be established. Third, the molecular targets and pathways identified through network pharmacology and molecular docking are predictive in nature and have not been experimentally validated in this study. Functional validation through gene silencing, pathway-specific inhibitors, or Western blot analysis of key hub genes would be necessary to establish causal relationships. Fourth, the selectivity of erucin toward cancer cells versus normal cells was not assessed, and future studies should include dose–response experiments using non-tumorigenic cell lines. Despite these limitations, the integration of computational and experimental approaches provides a coherent preliminary framework for understanding the potential biological effects of erucin in TNBC. Future studies should focus on pathway-specific validation, multi-cell-line analysis, and in vivo models to further substantiate these findings.

## 5. Conclusions

The present study provides preliminary evidence that erucin, a naturally occurring isothiocyanate derived from *E. sativa*, exhibits cytotoxic and antiproliferative effects against TNBC cells. Through in silico analyses, erucin was predicted to interact with multiple molecular targets associated with TNBC, including AKT1, STAT3, EGFR and MMP9. Functional enrichment analyses suggested potential associations with cancer related pathways, including EGFR tyrosine kinase inhibitor resistance, HIF-1 signaling, and estrogen signaling pathways central to TNBC pathogenesis. In vitro experiments demonstrate dose-dependent reduction in cell viability with an IC_50_ of 48.87 μg/mL MDA-MB-231 cells. Flow cytometry analysis showed increased apoptotic cell populations and G1 phase accumulation, indicating effects on cell survival and cell cycle distribution under the tested conditions. Taken together, these findings provide an initial systems-level perspective linking computational predictions with observed cellular responses. However, the molecular interactions and pathways identified in this study are predictive in nature and have not been experimentally validated. Therefore, further studies involving mechanistic validation, multiple TNBC cell lines and in vivo models are required to substantiate these observations and to better understand the potential role of erucin in TNBC.

## Figures and Tables

**Table 1:** Docking grid parameters used for each target protein.

Protein	PDB ID	Center (x, y, z)	Size (x, y, z)
AKT1	8UW7	14.852, 14.3528, −31.9736	66.22, 60.18, 55.15
STAT3	6NUQ	−2.2178, 19.1746, 24.6005	71.46, 115.04, 92.04
PTGS2	5KIR	31.3981, 8.0056, 35.307	78.49, 62.00, 63.39

**Table 2:** Binding affinities of erucin with target proteins.

Protein	PDB ID	Residue	Distance	Interaction Type	Binding Affinity (kcal/mol)
AKT1	8UW7	LEU 264 VAL 270 TYR 272 VAL 271	3.61 3.72 3.81 2.16	Hydrogen Bonds Hydrogen Bonds Hydrogen Bonds Hydrophobic Interactions	−7.5
STAT3	6NUQ	ASP 369 HIS 437	2.23 2.76	Hydrogen Bonds Hydrogen Bonds	−8.2
PTGS2	5KIR	HIS 122	3.38	Pi-Sulfur Interactions	−7.4

## Data Availability

All data generated or analyzed during this study are included in this article.
